# Validation of ESM1 Related to Ovarian Cancer and the Biological Function and Prognostic Significance

**DOI:** 10.7150/ijbs.66839

**Published:** 2023-01-01

**Authors:** Yu-kun Li, Tian Zeng, Yang Guan, Jue Liu, Nian-chun Liao, Meng-jie Wang, Ke-xin Chen, Xian-yu Luo, Chang-ye Chen, Fei-fei Quan, Juan Wang, Qun-feng Zhang, Juan Zou

**Affiliations:** 1 Department of Assisted Reproductive Centre, Zhuzhou central hospital, Xiangya hospital Zhuzhou central south university, Central South University, Zhuzhou, Hunan, China.; 2 Hunan Province Key Laboratory of Tumor Cellular & Molecular Pathology, Cancer Research Institute, University of South China, Hengyang, Hunan, China.; 3 School of Pharmacy, Jiangxi University of Chinese Medicine, Nanchang, Jiangxi, China.; 4 Department of Obstetrics and Gynecology, The Second Affiliated Hospital of University of South China, Hengyang, Hunan, China.; 5 Medical College, Hunan Polytechnic of Environment and Biology, Hengyang, Hunan, China.; 6 Department of gynecology, Clinical research center, The First Affiliated Hospital of Shenzhen University, Shenzhen Second People's Hospital, Shenzhen, Guangdong, China.; 7 Department of Pathology, Huizhou Sixth People's Hospital, Huizhou, Guangdong, China.; 8 Department of Obstetrics and Gynecology, Foshan First People's Hospital, Foshan, Guangdong, China.; 9 Center of Reproductive Medicine, The First-affiliated hospital of Hunan normal university, Hunan Provincial People's Hospital, Changsha, Hunan, China.

**Keywords:** Ovarian cancer, Bioinformatic analysis, ESM1, Akt/mTOR pathway, Prognostic marker

## Abstract

**Background:** Ovarian cancer (OC), a serious gynecological malignant disease, remains an enormous challenge in early diagnosis and medical treatment. Based on the GEO and TCGA databases in R language, endothelial cell-specific molecule 1 (ESM1) was confirmed separately with the bioinformatic analysis tool. ESM1 has been demonstrated to be upregulated in multiple cancer types, but the oncogenic mechanism by which ESM1 promotes OC is still largely unknown.

**Methods:** In this study, we used WGCNA and random survival forest variable screening to filter out ESM1 in OC differentially expressed genes (DEGs). Next, we confirmed the mRNA and protein levels of ESM1 in OC samples via PCR and IHC. The correlation between the ESM1 level and clinical data of OC patients was further confirmed, including FIGO stage, lymph node metastasis, and recurrence. The role of ESM1 in OC development was explored by several functional experiments *in vivo* and *in vitro*. Then, the molecular mechanisms of ESM1 were further elucidated by bioinformatic end experimental analysis.

**Results:** ESM1 was significantly upregulated in OC and was positively correlated with PFS but negatively correlated with OS. ESM1 knockdown inhibited cell proliferation, apoptosis escape, the cell cycle, angiogenesis, migration and invasion in multiple experiments. Moreover, GSVA found that ESM1 was associated with the Akt pathway, and our results supported this prediction.

**Conclusion:** ESM1 was closely correlated with OC development and progression, and it could be considered a novel biomarker and therapeutic target for OC patients.

## Introduction

Ovarian cancer (OC), a significant and global threat to women's lives, is one of the most lethal malignant tumors of the female reproductive system in the world 1. The survival time of patients with ovarian cancer has increased over the past 20 years with better surgery, more chemotherapy options, and various molecular targeting drugs. However, despite these advances, OC is still incurable in most patients in the late stage 2. Therefore, improving early detection, early diagnosis and timely, appropriate treatment are crucial for patients with OC 3. Especially in recent years, molecular targeting therapies have undergone several rapid and important developments. Indeed, some classic and famous molecular targets have been confirmed, including programmed cell death protein 1 (PD1) and epidermal growth factor receptor (EGFR) 4, 5. It has been validated that these molecular targets are effective and available in many OC patients but not in all OC patients because OC has large and different genetic mutation and epigenetic modification profiles 2. Molecular targeting drugs (gefitinib, dasatinib and erlotinib) in combination with carboplatin or paclitaxel show significant clinical efficacy for OC patients 6, 7. Molecular targeting drugs have achieved curative efficacy in OC. However, these molecular treatments are very expensive and difficult to carry out 2. Therefore, to better diagnose and treat OC patients, validation of biomarkers is needed. Many OC patients are already in the late stage at diagnosis, which increases the mortality of OC. The overall survival of late-stage patients is lower than that of early-stage patients 2, 3. Therefore, precise biomarkers for predicting prognosis and individualized therapy are needed. While encouraging, these results pointed out that OC-related precise biomarkers showed great diagnostic and prognostic potential, and they have not been systematically evaluated, resulting in constraining the potential of diagnostic markers and therapeutic targets in OC patients 8, 9. A deep understanding of the pathogenesis and molecular mechanism provides a new formulation strategy and experimental evidence for OC, which may contribute to improving the survival rate and early diagnosis rate.

With the rapid development of genomic microarrays and high-throughput sequencing technology combined with bioinformatics analysis, many analysis tools have been developed to explore available biomarkers, including weighted gene coexpression network analysis (WGCNA), the random survival forest algorithm 10 and random walking with restart (RWR) 8. WGCNA is a bioinformatic tool that can obtain hub genes related to the formation, development and progression of OC. This algorithm is widely used in exploring biomarkers at the transcriptional level. The random survival forest algorithm is an effective algorithm to identify the significance of prognostic biomarkers from numerous genes 10. In this study, two transcriptional level datasets based on the Gene Expression Omnibus (GEO) database were analyzed by WGCNA to validate differentially expressed genes (DEGs) between OC patients and normal women. Then, Gene Ontology (GO) and Kyoto Encyclopedia of Genes (KEGG) analyses were utilized to identify biological process, cellular component, molecular function, and pathway enrichment based on these DEGs. On the other hand, protein-protein interaction (PPI) network analysis was used to show all of the genes in the hub modules as central nodes. Furthermore, hub genes were also utilized in the random survival forest algorithm to gain correlations with the progression of OC. Subsequently, key genes based on these bioinformatic analyses were analyzed by a series of algorithms, such as GSVA, Immune correlation, Drug sensitivity, TMB, Oncoprint, and the Methylation and Genemania network. Eventually, we also experimentally identified the function of Endocan (ESM1) in the carcinogenesis of OC.

**Figure 1:**
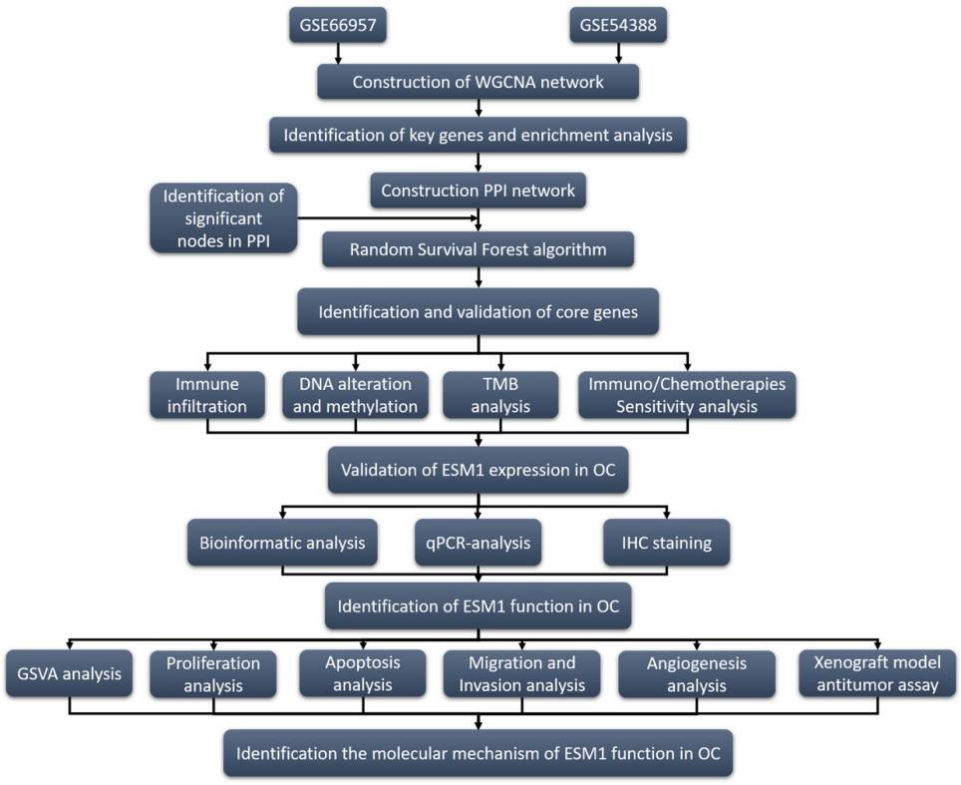
Program flowchart for this study.

ESM1, located on 5q11.2, is mainly expressed in endothelial cells in human lung and kidney tissues and is a significant index in clinic treatment, especially in angiogenesis 11. Previous studies indicated that ESM1 was physiologically expressed in the liver, lymph nodes, kidney, lung, skin and thyroid gland 12-14. It is also worth noting that the ectopic expression of ESM1 was confirmed in bladder urothelial carcinoma (BLCA), breast invasive carcinoma (BRCA), colon adenocarcinoma (COAD), cholangiocarcinoma (CHOL), lung adenocarcinoma (LUAD), esophageal carcinoma (ESCA), head and neck squamous cell carcinoma (HNSC), kidney renal clear cell carcinoma (KIRC), kidney chromophobe (KICH), liver hepatocellular carcinoma (LIHC), prostate cancer (PRAD), stomach adenocarcinoma (STAD), thyroid carcinoma (THCA), and uterine corpus endometrial carcinoma (UCEC) 15-18. ESM1 is involved in the progression of multiple cancers, including proliferation, migration, invasion, drug resistance, angiogenesis, and apoptosis escape 13, 18, 19. ESM1 could also regulate these molecular signaling pathways in cancer development and progression, such as the AKT/NF-kappaB/Cyclin D1 pathway 20, Wnt/β-catenin pathway 18, DLL4-Notch pathway 21, AKT/eNOS 22 and NFkB/iNOS signaling 22. To further elucidate the role of ESM1 in the development and progression of OC, we preliminarily explored its biological role in OC. The research strategy of this study is shown in Fig. 1.

## Materials and Methods

### GEO Data download

The Series Matrix File of GSE66957 was downloaded from the NCBI GEO public database. The expression profile data of 69 of ovarian cancer patients (n=12 for the normal group, n=57 for the tumor group) were obtained. The Series Matrix File of GSE54388 was downloaded, and the expression profile data of 22 of ovarian cancer patients (n=6 in the normal group, n=16 in the tumor group) were used for subsequent verification. The limma package was used for variance analysis, and the variance analysis filter condition was | logFC | > 1 & p < 0.05.

### WGCNA

By constructing the weighted gene coexpression network, the coexpression gene modules were found, and the association between the gene network and phenotype was explored, as well as the core genes in the network. The WGCNA-R packet was used to construct the coexpression network of all genes in the dataset, and the top 5000 genes with variance were screened by this algorithm for further analysis. The soft threshold of GSE66957 was set to 2, and the soft threshold of GSE54388 was set to 7. The weighted adjacency matrix was transformed into a topological overlap matrix (TOM) to estimate the network connectivity, and the hierarchical clustering method was used to construct the cluster tree structure of the TOM matrix. Different branches of the cluster tree represent different gene modules, and different colors represent different modules. Based on the weighted correlation coefficient of genes, the genes were classified according to their expression patterns, the genes with similar patterns were grouped into a module, and tens of thousands of genes were divided into multiple modules through gene expression patterns.

### TCGA data acquisition

The TCGA database (https://portal.gdc.cancer.gov/) is the largest cancer gene information database, including gene expression data, miRNA expression data and copy number variation, DNA methylation, SNPS and other data. We downloaded ovarian cancer data and raw mRNA expression data. A total of 379 cancer specimens were collected. GETx database (https://www.gtexportal.org/) is used to download normal ovary raw mRNA expression data, which included 88 normal ovary samples.

### Random survival forest variable screening

Random survival forest is a machine learning method to process survival data, and the tree building rules are similar to random forest. First, a univariate Cox proportional regression model was used to screen the prognostic genes in the training set. Bootstrap samples were randomly selected to construct 1000 classification trees. Then, in the process of tree building, at each node, some prediction variables are randomly selected as candidate variables to classify the node according to the survival criteria, including survival time and truncation information. Genes were screened for variables entering the model using exponential sequencing or frequency of gene occurrence. Every decision tree in the random survival forest is a dichotomous survival tree, and model overfitting can be avoided.

### Analysis of immune cell infiltration

The CIBERSORT method is a widely used method for evaluating immune cell types in the tumor microenvironment. Based on the principle of support vector regression, the expression matrix of immune cell subtypes was deconvoluted. It contains 547 biomarkers that distinguish 22 human immune cell phenotypes, including T cell, B cell, plasma cell, and myeloid cell subsets. In this study, the CIBERSORT algorithm was used to analyze the data of ovarian cancer patients to infer the relative proportion of 22 kinds of immune-infiltrating cells, and Spearman correlation analysis was performed on gene expression and immune cell content.

### Drug sensitivity analysis

Genomics database based on the largest drug (GDSC cancer drug sensitivity genomics database, https://www.cancerrxgene.org/), we used the R software package “pRRophetic” to predict the chemotherapy sensitivity of each tumor sample. Estimates of IC50 for each particular chemotherapeutic agent were obtained by regression, and the regression and prediction accuracy were tested by 10 cross-validation tests using the GDSC training set. Default values were selected for all parameters, including “combat” to remove batch effects and the average of repeated gene expression.

### GeneMANIA analysis

Genemania (http://www.genemania.org) is a flexible, user-friendly PPI network building database for visualizing functional networks between genes and analyzing gene function and interactions. The website can set the data sources of gene nodes, including physical interaction, gene coexpression, gene colocalization, gene enrichment analysis and website prediction. In this study, a core gene network was generated through GeneMANIA to explore the possible mechanism of its action in ovarian cancer patients.

### TMB analysis

The TMB data is download from TCGA. TMB is defined as the total number of somatic gene coding errors, base substitutions, insertions, or deletions detected per million bases. In this study, TMB was defined by dividing the non-synonymous mutation sites by the total length of the protein-coding region by calculating the variation frequency and variation/exon length of each sample.

### Cell culture

Ovarian cancer cells (CAOV4, OV90, SKOV3, CAOV3 and A2780), normal ovary cells (HOSE) and human umbilical vein endothelial cells (HUVECs) were purchased from the American Type Culture Collection (ATCC, Manassas, VA, USA). These cells were maintained in RPMI 1640 medium (Sigma‑Aldrich; Thermo Fisher Scientific, Inc.) (HOSE, A2780, SKOV3) or DMEM (Sigma‑Aldrich; Thermo Fisher Scientific, Inc.) (OV90, CAOV3, CAOV4) with 10% fetal bovine serum (FBS) (FBS; Gibco; In*vitro*gen; Thermo Fisher Scientific, Inc.), 100 IU/mL penicillin, and 10 µg/mL streptomycin (Thermo Fisher Scientific, Inc.). All cells were cultured at 37 °C with 5% CO2.

### Cell transfection and drug treatment

ESM1 shRNA was synthesized by HonorGene (Changsha, China). The target sequences were as follows: shESM1: 5'-TGGCATCTGGAGATGGCAATA-3'. For cell transfection, the ESM1 shRNA and vector/scramble shRNA plasmids were transfected into SKOV3 and A2780 cell lines at 37 °C for 24 h via Lipofectamine® 3000 (Thermo Fisher Scientific, Inc.). These shRNAs were transfected into the A2780 and SKOV3 cell lines with Lipofectamine 3000 (In*vitro*gen) according to the manufacturer's instructions. Human ESM-1 cDNA (NM_007036) was PCR-amplified from the cDNA library and cloned into the EcoRI/HindIII site of pcDNA3.1 (In*vitro*gen, Carlsbad, CA). Plasmids containing the ESM-1 coding region or pcDNA3.1 mock vector were transfected into the CAOV3 cell line with Lipofectamine 3000 (In*vitro*gen) according to the manufacturer's instructions. CAOV3 cells were treated with 10 µM LY294002 to inhibit the Akt pathway. SKOV3 and A2780 cells were treated with 4 µg/mL SC-79 to activate the Akt pathway.

### Clinical sample acquisition

A total of 60 ovarian cancer paraffin samples, 37 paracancer tissue paraffin samples, 15 normal ovarian paraffin samples, 40 fresh ovarian cancer tissues and 20 fresh normal ovarian tissues were collected from The First Affiliated Hospital of Shenzhen University (Shenzhen, Guangdong, China) and The Second Affiliated Hospital of University of South China (Hengyang, Hunan, China) from 2015 to 2020. The ethics standards were formulated in the Helsinki Declaration for these collections and the use of samples. Written informed consent was obtained from each patient, which was approved by the research ethics committee of University of South China and the research ethics committee of Shenzhen University.

### Proliferation analysis

For the MTT assay, 5000 cells were seeded into 96-well plates for culturing for 24, 48 and 72 hours at 37 °C with 5% CO2. Then, 20 µl of MTT solution (5 mg/ml, Sigma-Aldrich; Merck KGaA) was added to the bottom of the 96-well plates for 4 hours at 37 °C with 5% CO2. Then, 150 µl of DMSO was used to dissolve the precipitates, and the OD value was measured by a microplate reader (Molecular Devices, LLC) at 490 nm. For the colony formation assay, the cells of each group in the logarithmic growth phase were digested with 0.25% trypsin and blown into individual cells, and the cells were suspended in DMEM or 1640 culture medium of 10% fetal bovine serum for reserve. The cell suspension was diluted by gradient multiple, and each group of cells was inoculated with a gradient density of 50 cells in each dish into a dish containing 2 mL of preheated culture medium at 37 °C. The cells were gently rotated to disperse evenly. The cells were cultured at 37 °C in a cell incubator with 5% CO2 and saturated humidity for 14 days. For the EdU assay, operations were carried out according to EdU kit instructions (RiboBio, Guangzhou, China).

### FACS analysis

For the apoptosis assay, 3×10^5^ cells in 500 µl of binding buffer were incubated with 5 µl of Annexin V-APC and 5 µl of propidium iodide (KeyGEN Biotech, Nanjing, China) for 10 min. Then, flow cytometry was used to measure cell apoptosis. For the cell cycle assay, the cells were immobilized with ethanol and centrifuged at 800 rpm for 5 min, and the supernatant was discarded. The cells were cleaned twice with PBS. Then, 150 µl of PI (propidium iodide) (KeyGEN Biotech, Nanjing, China) working solution was added to these cells for 30 min at 4 °C. Next, flow cytometry was used to measure the cell cycle.

### Migration and invasion assay

For the wound healing assay, 5×10^5^ cells were seeded in 6-well plates, which were scratched with a pipette tip and washed with 1640/DMEM. Next, these cells were cultured at 37 °C for 24 h. The photographs were taken at 0 and 24 hours. For the Transwell assay, 2.5×10^5^ cells were seeded in a 24-well Transwell chamber (Costar, Cambridge, MA) with or without Matrigel for the invasion assay or migration assay and were cultured in 1640 with 10% FBS for 16 hours at 37 °C with 5% CO_2_. Finally, these migrating or invading cells at the lower surface of the filter were stained with crystal violet.

### Tube formation analysis

Matrigel (200 µl) was added to each well of a 48-well plate. A total of 1×10^4^ primary HUVECs in 50 µl of conditioned medium derived from A2780 and SKOV3 cells were seeded. After incubation at 37 °C for 6 h, images were collected using a fluorescence microscope.

### The zebrafish model

Transgenic zebrafish embryos with Tg(FLK1 :EGFP) at 48 HPF were selected and microinjected with about 600 OC cells/embryos (staining by CellTracker™ CM-DiI 1 ug/uL) to establish the zebrafish transplantation model of tumor cell line. After transplantation, the zebrafish was cultured in a 34 °C light incubator (14 hours of light and 10 hours of dark). Twenty-four hours after transplantation, juvenile fish with the same number of tumor cells were selected and cultured. Two days after injection, photographs were taken with a laser confocal microscope.

### Chorioallantoic membrane assay (CAM)

Firstly, the fertilized chicken eggs were maintained in an 80% humidified atmosphere at 37 °C for 7 days. In the day 8, a square window was cut on the shell to expose the CAM and was covered with a gelatin sponge (0.3 cm × 0.3 cm × 0.3 cm) containing PBS or the indicated conditioned medium (CM). Then, 2×10^5^ OC cells/100μl with Matrigel were placed onto one egg of chick embryo for 4 days. Finally, tape was used to cover the window for further incubation. After 2 days, angiogenesis was quantified by counting the number of blood vessel branches.

### Xenograft assay

Female athymic BALB/c nude mice (4 weeks old) were subcutaneously injected with 1×106 control and ESM1-shRNA1-A2780 cells. The tumor volume (cm^3^) was measured every seven days. The standard formula was width^2^ × length × 0.5. These nude mice were sacrificed at 48 days. Next, the xenografts were removed and weighed. Finally, these xenograft samples were dehydrated, embedded in paraffin, and sectioned for staining.

### qRT-PCR analysis

The method was described in our previously published papers 23. These RNAs were extracted by TRIzol® (In*vitro*gen; Thermo Fisher Scientific, Inc.) and reverse transcribed by HiScript III RT SuperMix for qPCR (Vazyme Biotech). The ESM1 primer sequences were as follows: ESM1 Forward: 5'-AGCTGGAATTCCATGAAGAG-3' Reverse: 5'-TCTCTCAGAAGCTTAGCCG-3'; GAPDH Forward: 5'-GAGTCAACGGATTTGGTCGT-3' Reverse: 5'-GATCTCGCTCCTGGAAGATG-3'.

The PCR parameters for cycling were as follows: 95 °C for 5 min, 50 cycles for 30 seconds, and 1 min at 60 °C. Each sample was carried out in 3 separate reactions, and the mean and standard error were calculated for each point. The expression levels of mRNA were normalized against the levels of GAPDH.

### IHC staining

The method was described in our previous publications 24. The primary antibodies used were as follows: ESM1 (Abcam, ab224591 at 1/100 dilution), VEGFA (Abcam, ab52917 at 1/100 dilution), PCNA (Abcam, ab265609 at 1/200 dilution), and Cdc25A (Abcam, ab2357 at 1/100 dilution).

### Western blot analysis

The method was described in our previous publications 23. The primary antibodies used were as follows: ESM1 (Abcam, ab103590 at 1/800 dilution), Akt (Abcam, ab8805 at 1/500 dilution), p-AKT (Abcam, ab38449 at 1/1000 dilution), mTOR (Abcam, ab245370 at 1/2000 dilution), p-mTOR (Abcam, ab109268 at 1/2000 dilution), MMP9 (Abcam, ab76003 at 1/2000 dilution), TIMP (Abcam, ab211926 at 1/1000 dilution), eNOS (Abcam, ab252439 at 1/1000 dilution), HIF-1α (Abcam, ab51608 at 1/500 dilution) and GAPDH (Abcam, ab181602 at 1/10000 dilution).

### Statistical analysis

All statistical analyses were performed using R language (Version 3.6). All statistical tests were bilateral, and P <0.05 was considered statistically significant.

## Results

### Identification of DEG sets associated with OC patients compared to normal women

**Figure 2:**
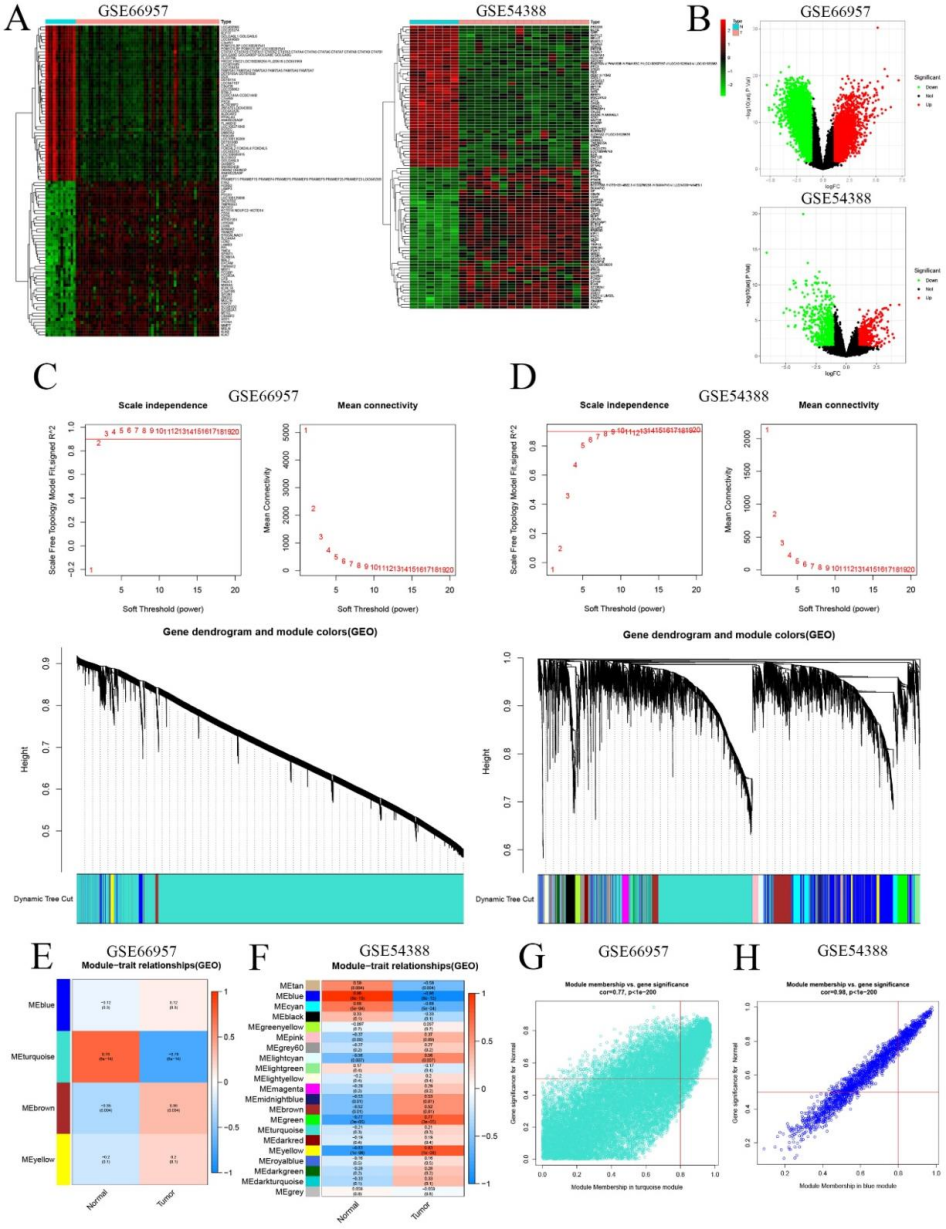
** Validation of the hub module via weighted gene coexpression network analysis (WGCNA).** DEGs are shown on the heatmap **(A)** and the volcano plot **(B)** for the GSE66957 and GSE54388 datasets. The scale-free fit index and the average connectivity of soft threshold power and hierarchical clustering tree of genes based on topological overlap are confirmed for GSE66957 **(C)** and GSE54388 **(D)**. The correlation of these modules between the normal group and tumor group for GSE66957 **(E)** and GSE54388 **(F)**. A scatter plot of the turquoise module for GSE669857 **(G)** and the blue module for GSE54388 **(H)**.

First, we extracted the RNA level profiles of two datasets, GSE66957 (57 OC samples and 12 normal ovary samples) and GSE54388 (16 OC samples and 6 normal ovary samples), from the GEO database for subsequent validation. To characterize the transcriptome of OC patients, we screened genes that were differentially expressed (|logFC|>1 & p<0.05) (Figure 2A&B). Furthermore, we used WGCNA to construct the gene coexpression network of OC based on the GSE66957 and GSE54388 datasets. We selected β = 2 for GSE66957 and β = 7 for GSE54388 to construct a scale-free network (Figure 2A, 2B). Subsequently, we used dynamic hybrid cutting to construct a hierarchical clustering tree that made a gene module. The tree branch showed several genes with analogous expression profiles. Each single gene represented a leaf on the tree (Figure 2C, 2D). Moreover, four modules were constructed for GSE66957, and twenty-one modules were constructed for GSE54388 (Figure 2E, 2F). According to the cutoff standard (Module-Membership = 0.77 and Gene-Significance < 1e-200 for GSE66957, and Module-Membership = 0.98 and Gene-Significance < 1e-200 for GSE54388), the turquoise and blue modules were identified as candidate hub modules for the GSE66957 and GSE54388 datasets, respectively (Figure 2G, 2H).

### Identification of key genes and enrichment analysis

To validate the reliability of key genes, we utilized a Venn analysis using the data of DEG sets and hub gene sets based on the GSE66957 and GSE54388 datasets, respectively (Supplement Figure 1A). A total of 326 genes were screened to perform GO and KEGG enrichment analyses. GO analysis indicated that these genes were enriched in multiple biological process terms, including cell-cell adhesion via plasma-membrane adhesion molecules, organic acid catabolic process and carboxylic acid catabolic process, in multiple cellular component terms, including the apical part of the cell, the cell-cell junction, and the apical plasma membrane, and in multiple molecular function terms, including coenzyme binding, serine-type peptidase activity, and hydrolase activity, acting on acid phosphorus-nitrogen bonds (Supplement Figure 1B). KEGG analysis showed that tight junctions, pathogenic Escherichia coli infection, and the Wnt signaling pathway were enriched by these key genes (Supplement Figure 1C). Moreover, we built a PPI network for these key genes, as shown in Supplement Figure 1D.

### Identification and validation of core genes

**Figure 3:**
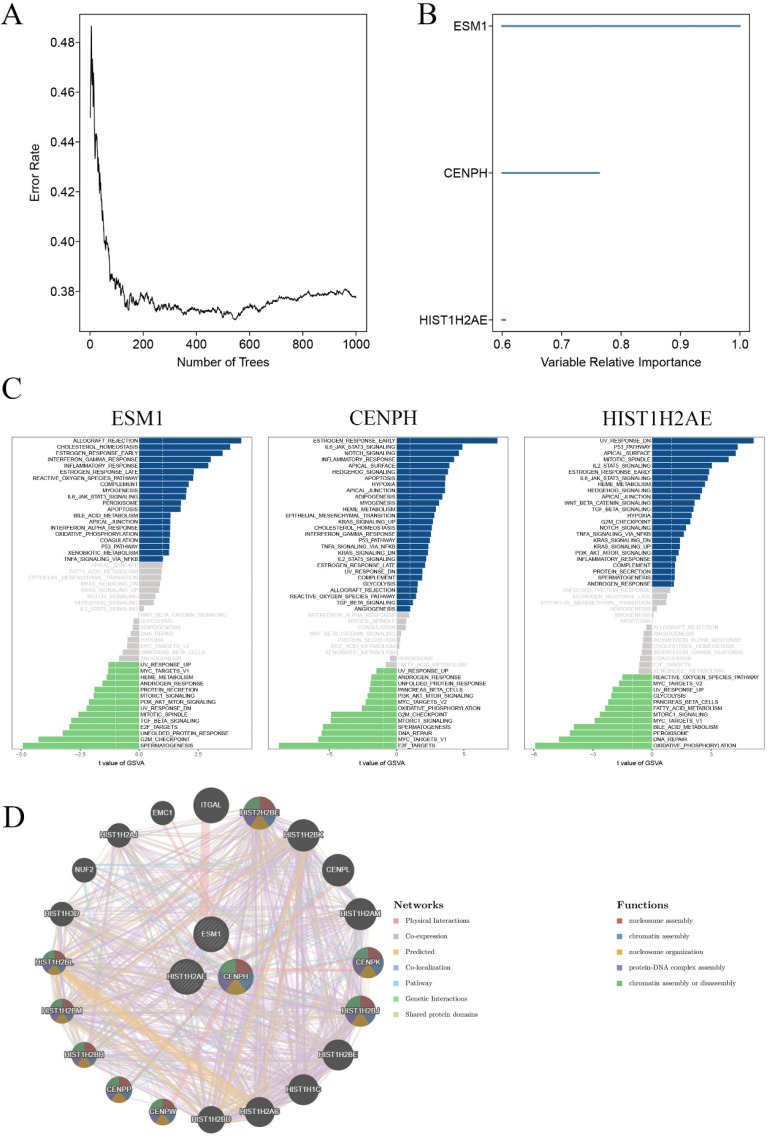
** Validation of key genes in OC. A.** Correlation between the error rate and number of trees. **B.** Variable relative importance of the sequencing of three genes. **C.** GSVA of three key genes, ESM1, CENPH and HIST1H2AE. **D.** The networks among ESM1, CENPH and HIST1H2AE based on the GeneMANIA database.

To identify core genes for the carcinogenesis of OC, we then used the random survival forest algorithm for feature selection. The relationship between the error rate and the number of taxonomic trees was used to reveal genes with relative importance greater than 0.38 as the final signature (Figure 3A). Finally, we validated 3 genes (CENPH, ESM1 and HIST1H2AE). To test the property of the random survival forest model on these data, we determined the out-of-bag (OOB) error, which was analyzed by testing each tree against data not utilized in building that tree. The significant order of the OOB scores for the 3 genes is shown in Figure 3B. Furthermore, gene set variation analysis (GSVA) suggested that the estrogen response was early, and E2F targets were the main pathway mediated by CENPH. ESM1 primarily mediated allograft rejection and the spermatogenesis pathway. UV response dn and oxidative phosphorylation principally served as one of the two major enriched pathways with HIST1H2AE expression (Figure 3C). Furthermore, the Genemania network also indicated that the three genes have a close interaction in multiple biological functions, including nucleosome assembly, chromatin assembly, nucleosome organization, and protein-DNA complex assembly (Figure 3D).

### Core genes related to immune infiltration and genetic alterations in OC

Core genes were validated by a series of bioinformatic analyses, such as WGCNA, GO, KEGG, and random survival forest. This indicated that these three genes were involved in OC progression. Therefore, we further defined the three genes CENPH, ESM1 and HIST1H2AE as the core genes associated with OC. For further identification, as shown in Supplement Figure 2A, we also extracted clinical association data of these 3 genes based on the TCGA database. This result showed that the expression of ESM1 increased in pathological grade 2 OC tissue compared to pathological grade 3 OC tissue. However, the expression of CENPH and HIST1H2AE had no significant correlation with pathological grade in OC (Supplement Figure 2A). To elucidate the relationship between the immune microenvironment and the three genes, we extracted the level profiles of the three genes based on the TIMER database, which indicated different expression levels in different immune cell types (Supplement Figure 2B). Furthermore, we further analyzed the association of the levels of the three genes with the infiltration levels of B cells, CD8^+^ T cells, CD4^+^ T cells, macrophages, neutrophils and dendritic cells (Supplement Figure 2C). Therefore, these results indicated that the three genes were intimately correlated with the immune infiltration progression of OC patients, especially in HIST1H2AE and ESM1.

**Figure 4:**
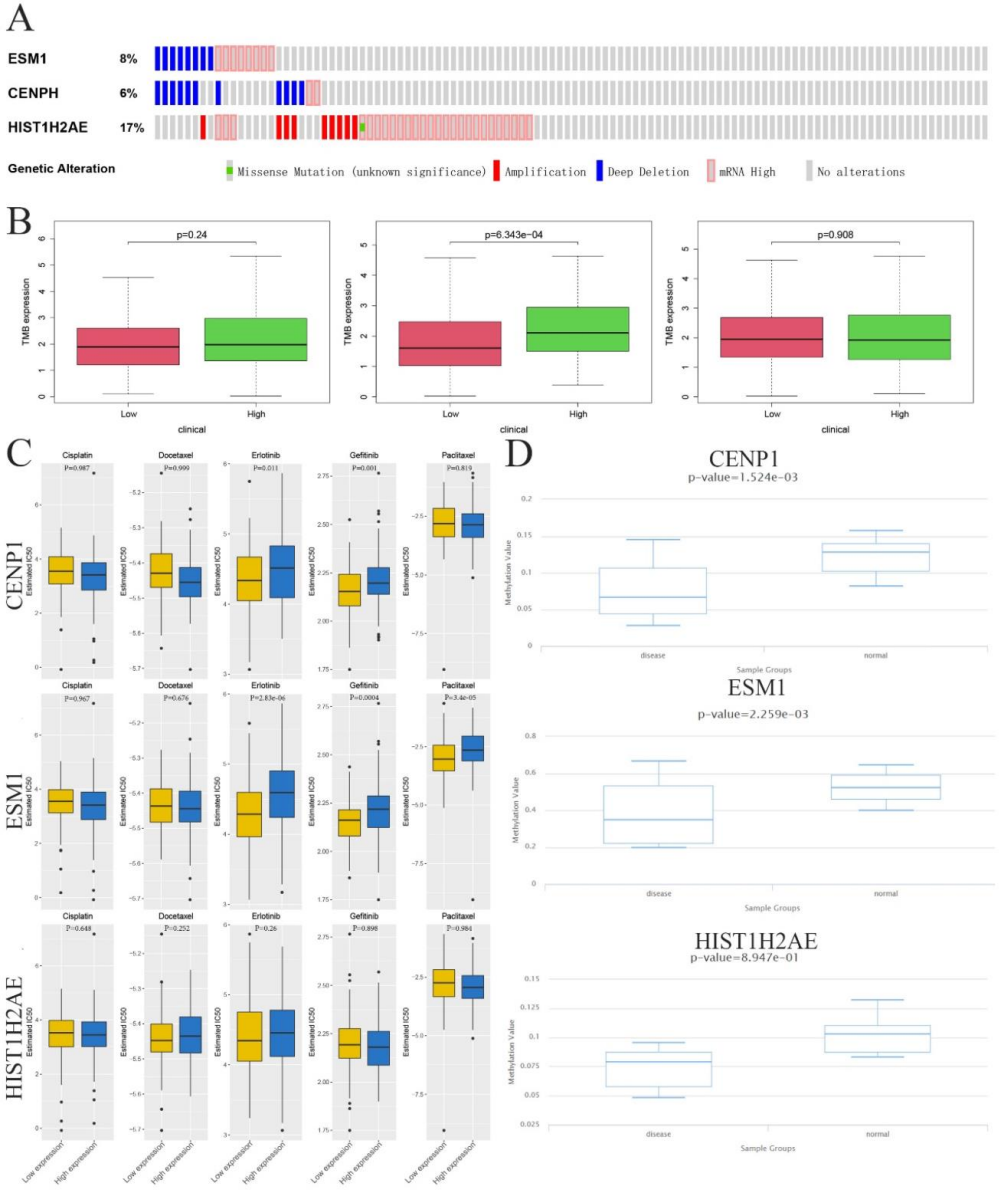
** Epigenetic modification and genetic alteration of 3 key genes. A.** The DNA alteration of 3 key genes. **B.** Tumor mutation burden of 3 key genes.** C.** The estimated IC_50 values_ of cisplatin, docetaxel, erlotinib, gefitinib and paclitaxel for 3 key genes. **D.** The DNA promoter methylation levels of 3 key genes based on the TCGA database.

To identify whether three genes exist in the somatic mutation frequencies in OC, we extracted the oncoprint profiles based on the cBioPortal database (http://www.cbioportal.org/). The somatic mutation frequencies for CENPH, ESM1 and HIST1H2AE were 6%, 8% and 17%, respectively (Figure 4A). Moreover, another analysis indicated a significant association between immune infiltration and carcinogenesis. Tumor mutational burden (TMB) is a markedly important and identifiable clinical biomarker for immunotherapy. To investigate whether TMB may influence immunotherapy in OC with ectopic expression of these genes, including CENPH, ESM1 and HIST1H2AE, we confirmed the correlation between the expression of these genes and the number of somatic mutations, which indicated that high expression of ESM1 was associated with a higher TMB than low expression of ESM1 in OC (p=6.343e-04) (Figure 4B). These results indicated that the immune checkpoint inhibitor might be more effective and sensitive for OC patients with high expression of ESM1.

### Sensitivity to Immuno/Chemotherapies for Core Genes in OC

Examining the TMB of three genes allowed us to analyze the possibility of efficiency of immunological therapy. Although immunological therapy is now not being used in patients with OC, we utilized the TIDE algorithm to seek the possibility of efficiency for immunological therapy. Considering the chemotherapies for use in routine work, we also assessed the response of two genetic expression subtypes (low or high expression level) to three chemotherapeutic drugs and two EGFR-TK inhibitors: cisplatin, docetaxel, paclitaxel, gefitinib and erlotinib. Therefore, we utilized a predictive model by ridge regression based on GDSC cell line data. Interestingly, this result showed the IC_50_ was between low and high level ESM1 for these five drugs where low ESM1 could be more sensitive to chemotherapies (paclitaxel) and immunological therapies (gefitinib and erlotinib). However, this difference was not observed in CENPH and HIST1H2AE after treatment with the five drugs (Figure 4C). Taken together, these results indicated that the OC patients with high level of ESM1 were better candidates for immunotherapy than EGFR-TK inhibitors (Erlotinib and Gefitinib) and paclitaxel. Moreover, a previous study indicated that aberrant epigenetic modification can drive tumorigenesis and resistance to treatment 25. We extracted the DNA methylation profiles of these core genes in OC based on the TCGA database, which showed that the methylation level was increased in OC compared to normal ovarian tissues (Figure 4D).

### Ectopic expression of ESM1 is correlated with clinical parameters in OC

**Figure 5:**
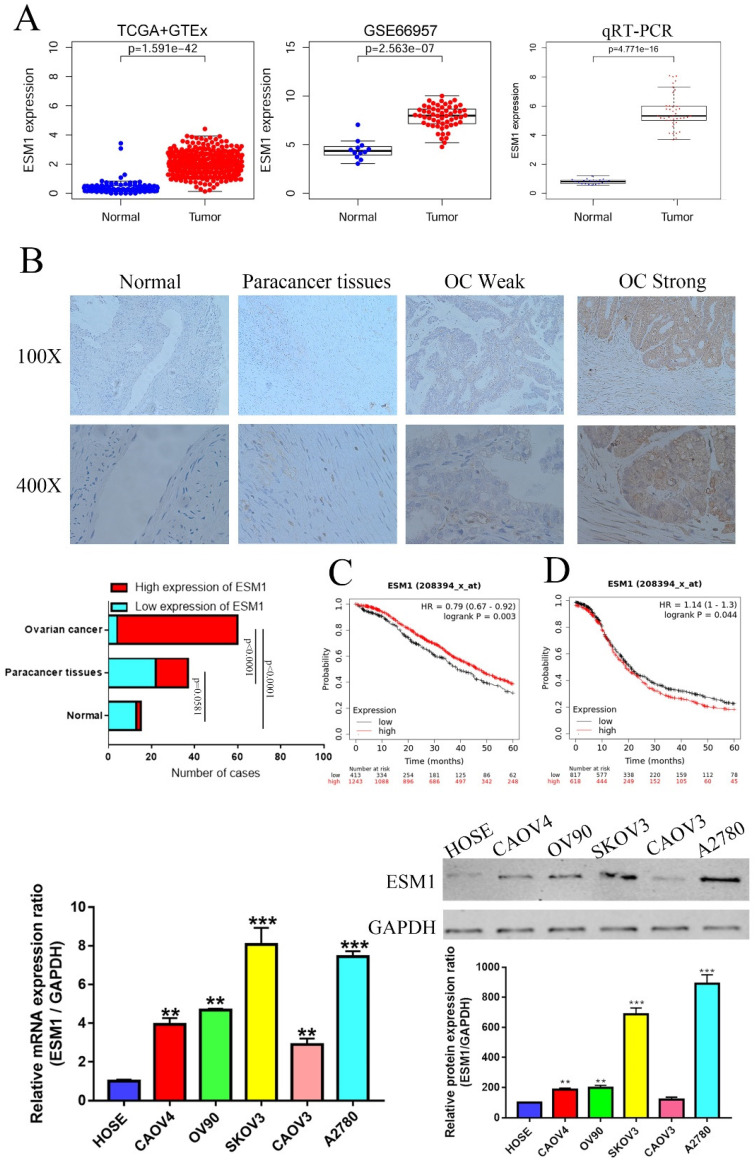
** The expression level and prognostic value of ESM1 in OC patients. A.** Identification of the mRNA level of ESM1 based on the TCGA database OC dataset, GEO database GSE66957 dataset and qRT-PCR in OC patients. **B.** The protein expression level of ESM1 in normal ovary tissue samples, paracancerous OC tissue samples and OC tissue samples. **C.** The OS analysis of ESM1 based on the KM-plot database. **D.** PFS analysis of ESM1 based on the KM plot database. **E.** The mRNA level of ESM1 in multiple OC cell lines and immortalized ovarian epithelial cells. **F.** ESM1 protein expression in multiple OC cell lines and immortalized ovarian epithelial cells. **P < 0.01, ***P < 0.001 indicates statistical significance compared with the control.

We first used the TCGA and GEO databases to confirm the expression of ESM1 in OC patients, indicating that ESM1 was obviously enhanced in OC (Figure 5A). To further study the mRNA levels of these core genes in OC, we collected 30 pairs of adjacent normal ovarian tissues from OC patients undergoing surgical lobectomies. qRT-PCR also showed that ESM1 was significantly increased in OC compared to corresponding paracancer tissues (Figure 5A). We also found that ESM1 had a highly sensitivity and specificity in OC diagnosis based on TCGA database (Supplement Figure 3A), which indicated that ESM1 was an excellent diagnostic marker for OC patients. Furthermore, we utilized IHC staining to detect these protein levels in OC compared to corresponding paracancerous tissues and normal ovarian tissues. As we predicted, the expression of ESM1 was increased in OC tissues compared to corresponding paracancerous tissues and normal ovarian tissues (Figure 5B). Furthermore, we also found that these ectopically expressed proteins were closely correlated with some clinical parameters of OC (Tables 1-2), such as the FIGO stage, lymph node metastasis, and recurrence. These results indicated that ESM1 might play key roles in the development and progression of OC.

We also validated the prognostic significance of ESM1 in OC patients based on the KM-plot database, which indicated that the overall survival (OS) of ESM1 levels was negatively correlated with OS patient prognosis (Figure 5C) but that the progression-free survival (PFS) of ESM1 levels was positively related to the prognosis of patients with OC (Figure 5D). Previous study indicated that the existence of intimate cross-talk between the hypertrophied heart and the tumor that is mediated by secreted factors, leading to cancer promotion and disease deterioration 26. This paradoxical result might be attributed to the fact that ESM1 could accelerate the progression of OC to negatively correlated PFS, but OC patients with high levels of ESM1 could benefit from cardiopulmonary failure 27, 28, which allowed OC patients with high ESM1 level to have a higher tolerance and thus avoid death from heart and lung failure. Moreover, we also found ESM1 was significantly increased in the OC patients with early stage (Supplement Figure 4), which indicated that more patients with early-stage OC patients with high ESM1 mRNA expression have a better clinical treatment opportunity to obtain a better prognosis compared to OC patients with low ESM1 mRNA expression. We further analyzed the prognostic value of ESM1 using TCGA database, as shown in Supplement Figure 3B-F. The ESM1 mRNA expression was significantly and positively correlated with overall survival, especially in these OC patients with lymphatic invasion, G3 histologic grade, Age>60, and FIGO stage III. The ESM1 expression was also higher in unilateral OC patients compared to bilateral OC patients (Supplement Figure 3G). In DSS and OS event, alive OC patients had higher ESM1 mRNA expression than these OC patients with dead event (Supplement Figure 3H). These results indicated that ESM1 had an important function in OC development and progression. Finally, we confirmed the ESM1 expression level in multiple OC cell lines. qRT-PCR and western blotting showed that the ESM1 mRNA and protein levels were significantly increased in OC cell lines compared to normal ovarian cells (HOSE), especially in SKOV3 and A2780 cells (Figure 5C&D).

### The effect of ESM1 knockdown on OC progression

**Figure 6:**
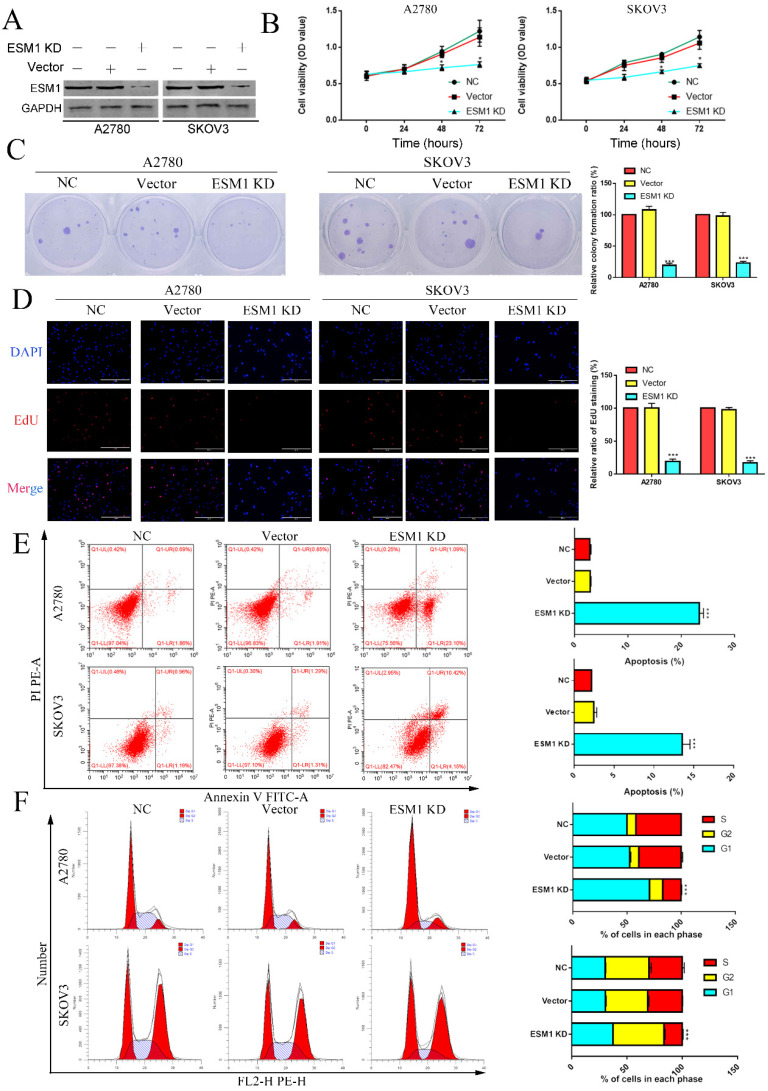
** The effect of ESM1 on OC proliferation, cell cycle progression and apoptosis. A.** The expression of ESM1 in the NC, vector and ESM1 KD groups of A2780 and SKOV3 cells. The effect of ESM1 KD on A2780 and SKOV3 cells via MTT analysis **(B)**, plate clone formation assay **(C)**, and EdU assay** (D)**. **E.** The function of ESM1 KD in OC cell apoptosis escape. **F.** The effect of ESM1 KD on the cell cycle in A2780 and SKOV3 cells. *P < 0.05, **P < 0.01, ***P < 0.001 indicates statistical significance compared with the control.

**Figure 7:**
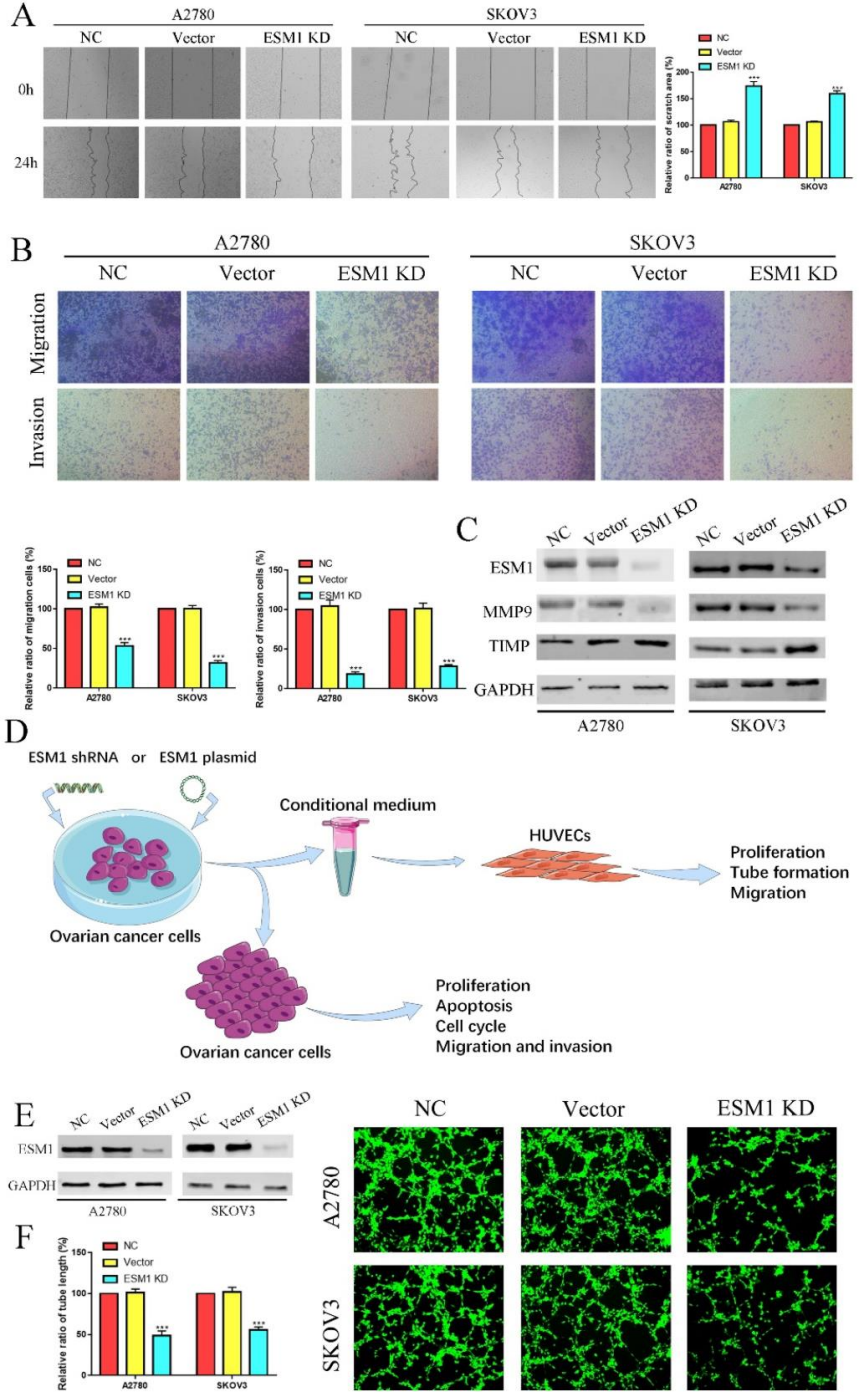
** ESM1 KD impedes OC migration, invasion and angiogenesis.** The migration and invasion ability of the NC, vector, and ESM1 knockdown were measured and compared in A2780 and SKOV3 cells by wound healing **(A)** and Transwell assays** (B)**. **C.** The level of MMP9 and TIMP of ESM1 KD by shRNA compared with the normal control and vector group by Western blotting in A2780 and SKOV3 cell lines. **D.** A workflow of cell transfection, conditional medium collection and analyses for tube formation and other experiments. **E.** The expression of ESM1 in the NC, vector and ESM1 KD groups of A2780 and SKOV3 cells. **F.** Cell angiogenesis assays were performed in A2780 and SKOV3 cells transfected with the vector or ESM1 shRNA. ***P < 0.001 indicates statistical significance compared with the control.

To validate the pathophysiological function of ESM1, we used RNA interference to inhibit endogenous ESM1 expression. Western blotting was used to confirm endogenous ESM1 expression, which showed that the best inhibitory effect of shRNA was shRNA#1 (Figure 6A). We confirmed the effects of ESM1 on OC proliferation, which indicated that inhibiting ESM1 could significantly reduce the proliferation ability *in vitro* (Figure 6B&C). Moreover, we found that ESM1 expression knockdown significantly repressed the DNA replication level in A2780 and SKOV3 cell lines (Figure 6D). These results indicated that ESM1 knockdown could significantly inhibit the proliferation ability of OC cells. Then, we confirmed the effect of ESM1 on apoptosis levels in A2780 and SKOV3 cells by flow cytometry. The results showed that the apoptosis level was obviously increased in two OC cell lines when ESM1 was deficient (Figure 6E). Moreover, ESM1 KD obviously induced cell cycle arrest at the G1 phase (Figure 6F). We also detected the effect of ESM1 knockdown on the migration and invasion of OC cells. OC migration and invasion were obviously attenuated by ESM1 inhibition (Figure 7A&B). ESM1 knockdown also inhibited MMP9 expression but augmented TIMP expression to suppress OC cell migration and invasion (Figure 7C).

### ESM1 deficiency represses OC angiogenesis *in vivo* and *vitro*

Furthermore, we used the conditional medium (CM) derived from OC cells after ESM1 KD or OE transfection to incubate HUVECs for 6 hours, which were utilized for further tube formation analysis, proliferation analysis, migration analysis (Figure 7D). The tube formation analysis showed that ESM1 deficiency could induce tumor neovascularization inhibition compared to the NC and vector groups (Figure 7E&F).

**Figure 8:**
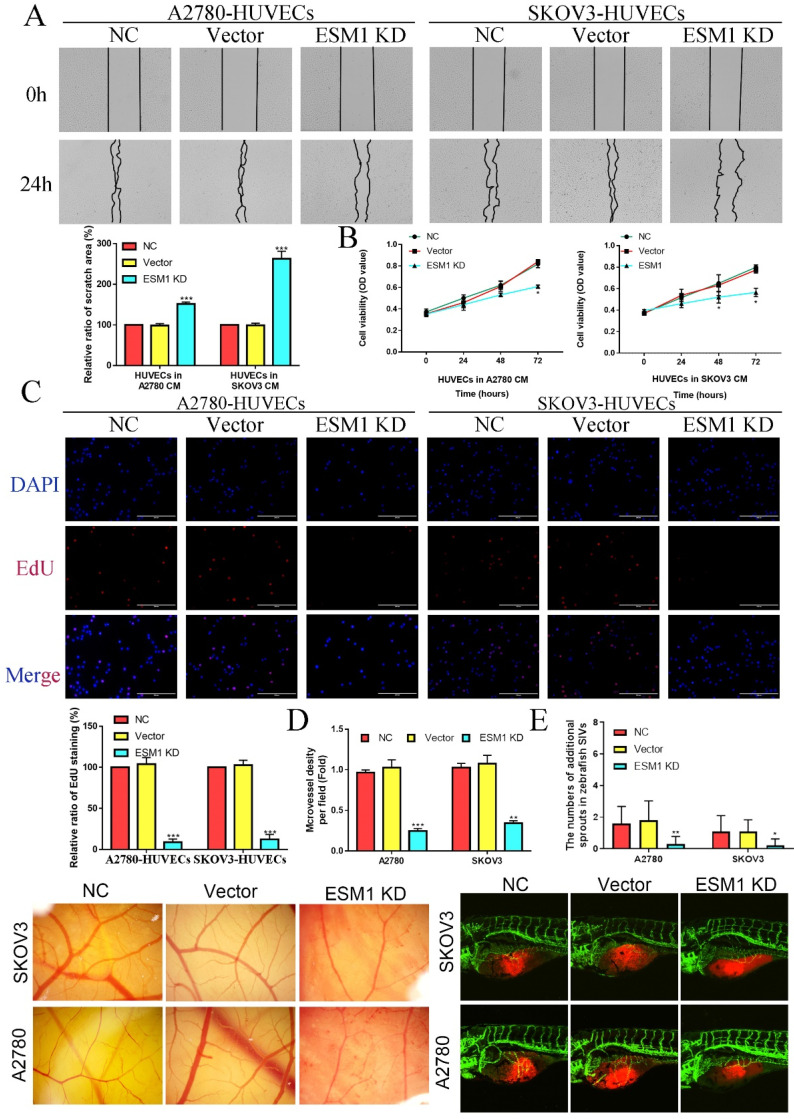
** ESM1 knockdown can repress OC cell angiogenesis *in vitro* and vivo. A.** A2780/SKOV3-conditioned medium from cells transfected with Vector or ESM1 shRNA enhanced the HUVECs migration ability by wound healing analysis. MTT analysis **(B)** and EdU assay** (C)** for the effects of OC cell conditioned medium with ESM1 KD on HUVECs proliferation. **D.** Chicken embryos were incubated with A2780/SKOV3-conditioned medium after ESM1 KD or not. **E.** The effects of ESM1 shRNA transfection or empty vector plasmid transfection on A2780/SKOV3 angiogenesis in zebrafish model. *P < 0.05, **P < 0.01, ***P < 0.001 indicates statistical significance compared with the control.

Wound healing assay showed that the migration ability of HUVECs was significantly decreased in the CM derived from OC cells after ESM1 KD (Figure 8A). The proliferation assay also indicated that the HUVECS with the CM derived from ESM1 KD OC cells had lower proliferation ability compared to NC and Vector group (Figure 8B&C). Moreover, CAM analysis was used to further confirm the molecular effects of ESM1 on OC angiogenesis *in vivo*, compared to NC and Vector group, ESM1 KD had an obviously decreased density of new blood vessels formation (Figure 8D). The effects of ESM1 inhibition was also observed in the zebrafish model, which showed ESM1 KD could significantly repress the formation of new blood vessels *in vivo* (Figure 8E). These results indicated that ESM1 knockdown could attenuate the angiogenesis ability of OC cells.

### The effect of ESM1 inhibition on tumor growth *in vivo*

**Figure 9:**
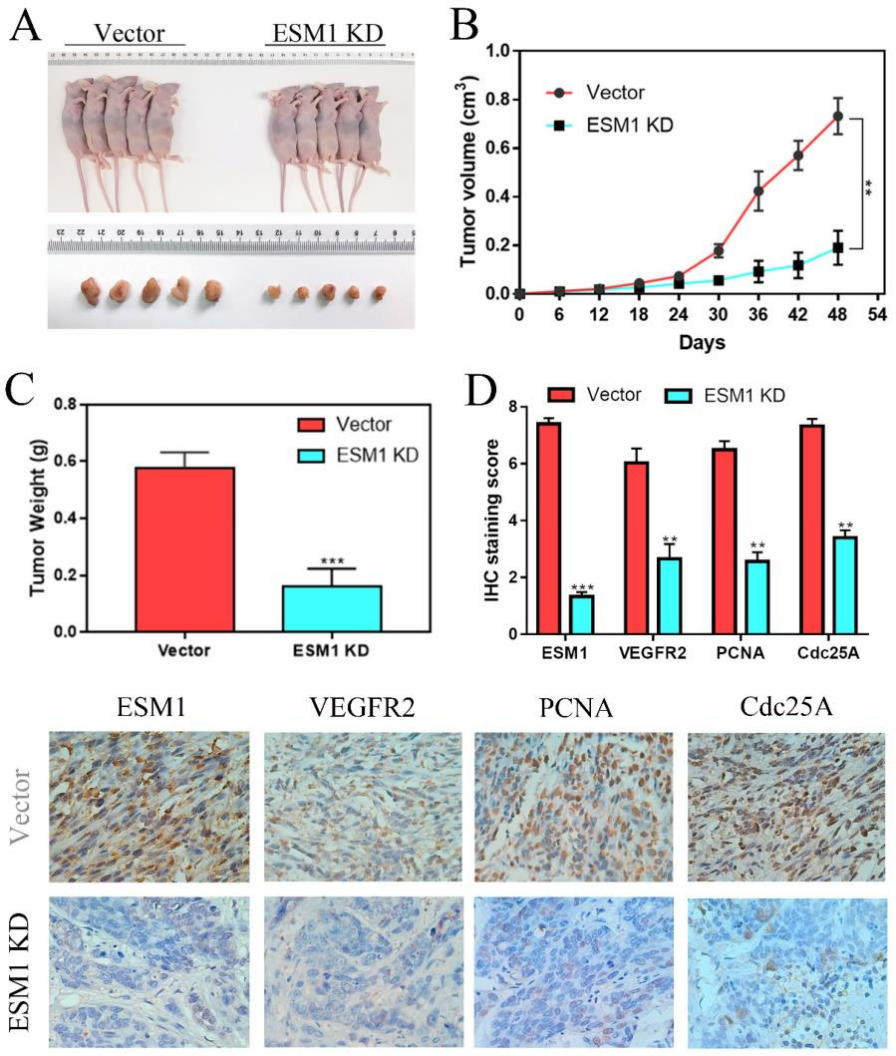
** ESM1 inhibition can repress OC cell growth *in vivo*. A.** The effects of ESM1 shRNA lentivirus plasmid transfection or empty vector lentivirus plasmid transfection on A2780 growth *in vivo*. **B.** The tumor volume and **C.** tumor weight of these xenografts. **D.** The expression of ESM1, VEGFA, PCNA and Cdc25A in these xenografts via IHC staining. **P < 0.01, ***P < 0.001 indicates statistical significance compared with the control.

To further confirm the effect of ESM1 on OC *in vivo*, A2780 cells transfected with vector or ESM1 KD were subcutaneously injected into nude mice. We found that tumor volumes and weights were obviously smaller in the ESM1 inhibition group than in the vector group (Figure 9A-C), which indicated that ESM1 could promote the tumorigenicity of OC cells *in vivo*. IHC staining showed that ESM1 inhibition could inhibit the expression of VEGF-A, PCNA and Cdc25A (Figure 9D), suggesting that ESM1 could accelerate OC progression by promoting proliferation, the cell cycle and angiogenesis.

### EMS1 knockdown represses the Akt/mTOR/HIFα pathway in human OC cells

**Figure 10:**
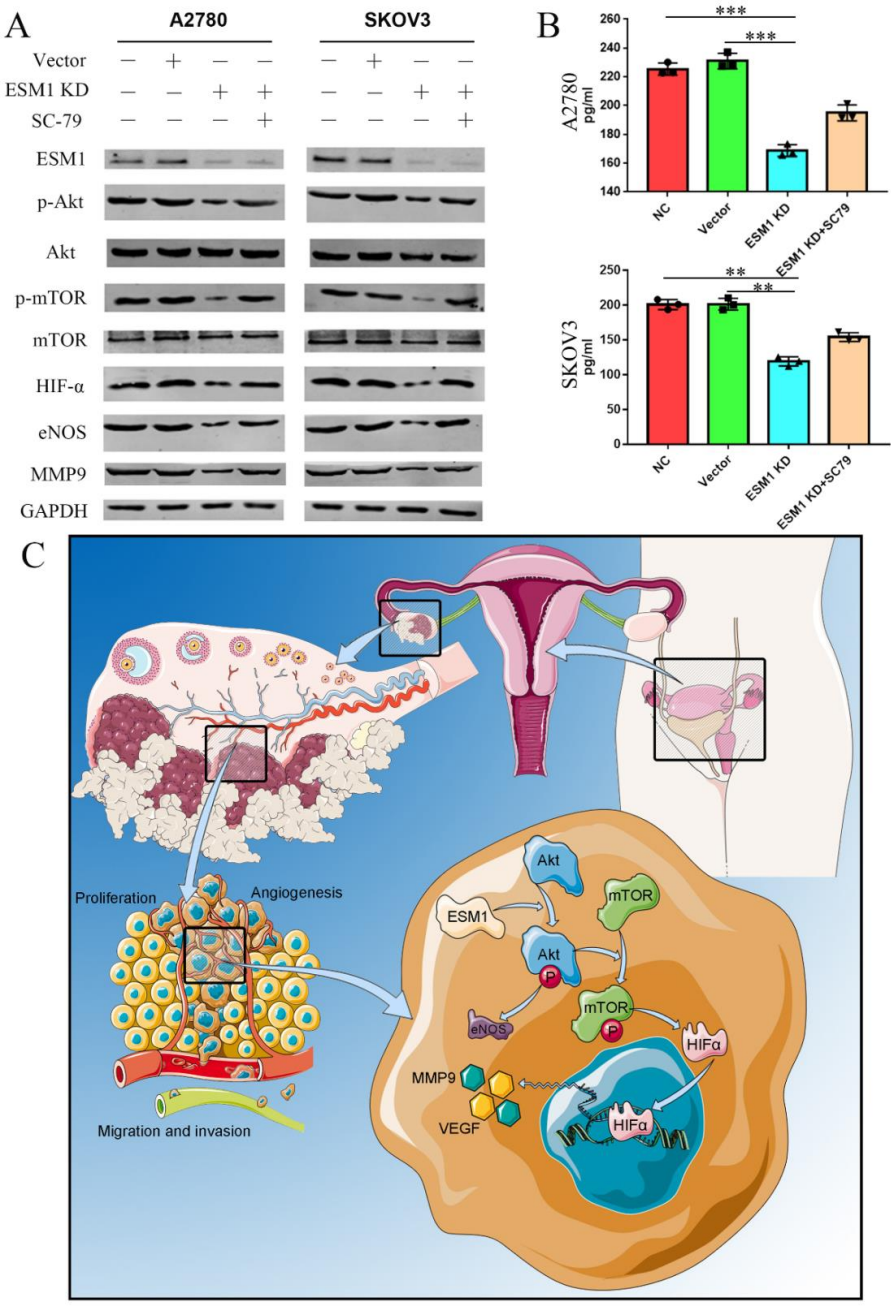
** ESM1 accelerates OC development and progression via the Akt/mTOR pathway. A.** The expression of ESM1, Akt, p-Akt, mTOR, p-mTOR, HIF-α, eNOS, and MMP9 was confirmed in the NC, vector, ESM1 KD and ESM1 KD with SC-79 (Akt pathway activator) groups by Western blotting.** B.** The level of VEGF was detected in the NC, vector, ESM1 KD and ESM1 KD with SC-79 (Akt pathway activator) groups by ELISA analysis. **C.** The potential mechanisms of ESM1 in OC. **P < 0.01, ***P < 0.001 indicates statistical significance compared with the control.

According to the GSVA results (Figure 3C), we confirmed the onco-genetic effects of ESM1 on the PI3K/Akt pathway. Western blot analysis showed that ESM1 inhibition could inactive the AKT pathway and further reduce the expression of Akt, p-Akt, mTOR, p-mTOR, HIF-α, eNOS and MMP9, which could be reversed by an Akt activator (SC-79) (Figure 10A). Furthermore, we detected the VEGF level in these cell groups by ELISA (Figure 10B), which indicated that ESM1 could increase the level of VEGF via the Akt pathway. In conclusion, the ESM1/Akt axis plays a key role in the regulation of OC cell proliferation and angiogenesis (Figure 10C).

### ESM1 overexpression (OE) accelerates proliferation, migration, and angiogenesis in OC cells via the Akt pathway

**Figure 11:**
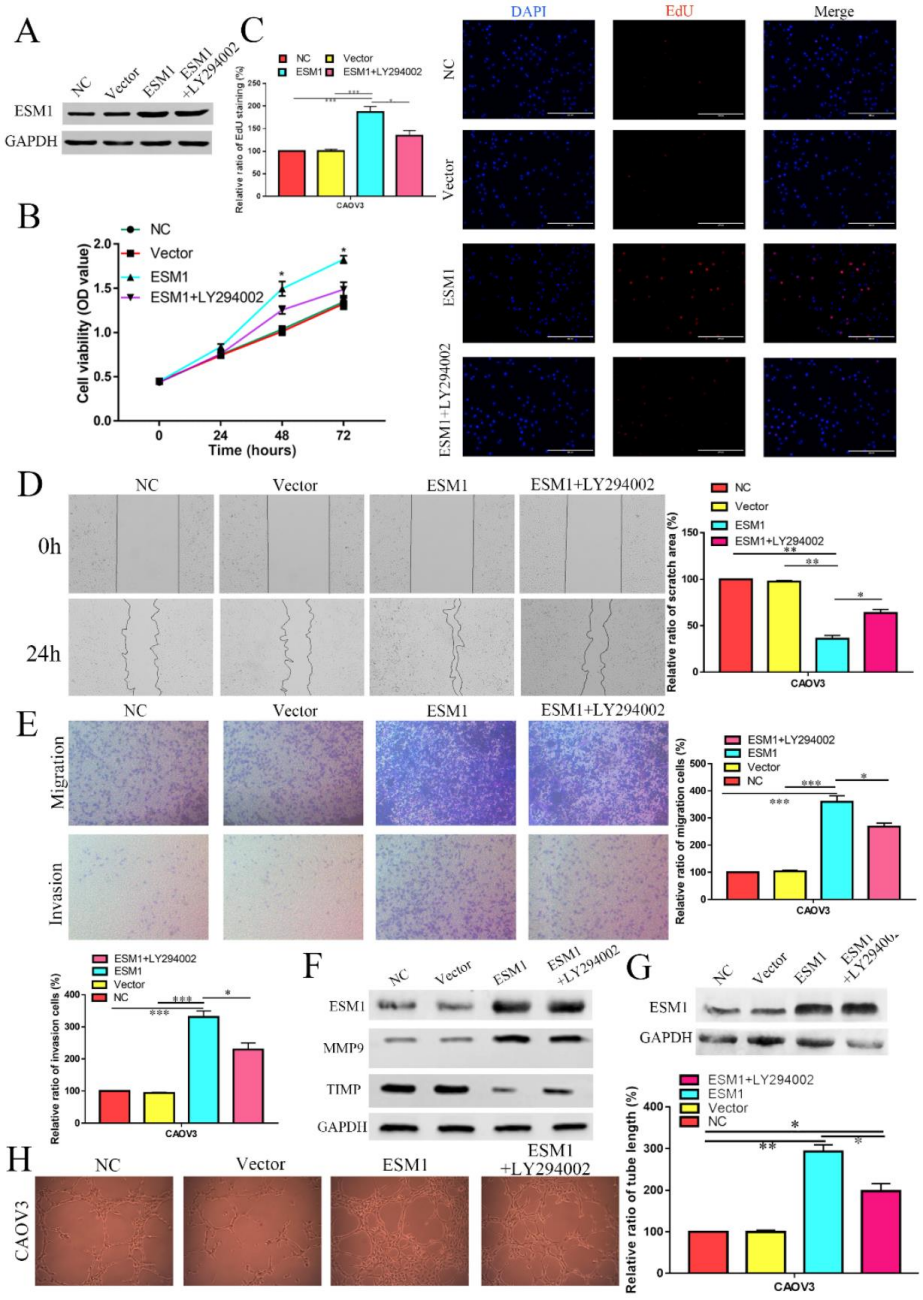
** The effect of ESM1 overexpression on the OC biological phenotype via the Akt pathway. A.** The expression of ESM1 in the NC, vector, ESM1, and ESM1+LY294002 groups in CAOV3 cells by Western blotting. The effect of ESM1 and ESM1+LY294002 on CAOV3 cell proliferation ability via MTT analysis **(B)** and EdU assay **(C)**. The effect of ESM1 and ESM1+LY294002 on CAOV3 cell migration and invasion ability via wound healing **(D)** and Transwell assays **(E)**. **F.** The levels of MMP9 and TIMP in ESM1 and ESM1+LY294002 cells compared with the normal control and vector groups by Western blotting in CAOV3 cell lines. **G.** The expression of ESM1 in the NC, vector, ESM1 and ESM1+LY294002 groups in CAOV3 cell lines. **H.** Angiogenesis assays were performed in CAOV3 cells transfected with vector, ESM1, or ESM1+LY294002. *P < 0.05, **P < 0.01, ***P < 0.001 indicates statistical significance compared with the control.

Moreover, we constructed a CAOV3 cell line that overexpressed ESM1 protein and an NC CAOV3 cell line that was transfected with the empty vector. The ESM1 level was detected by Western blotting (Figure 11A). MTT analysis showed that ESM1 OE could significantly increase the viability of CAOV3 cells (Figure 11B).

**Figure 12:**
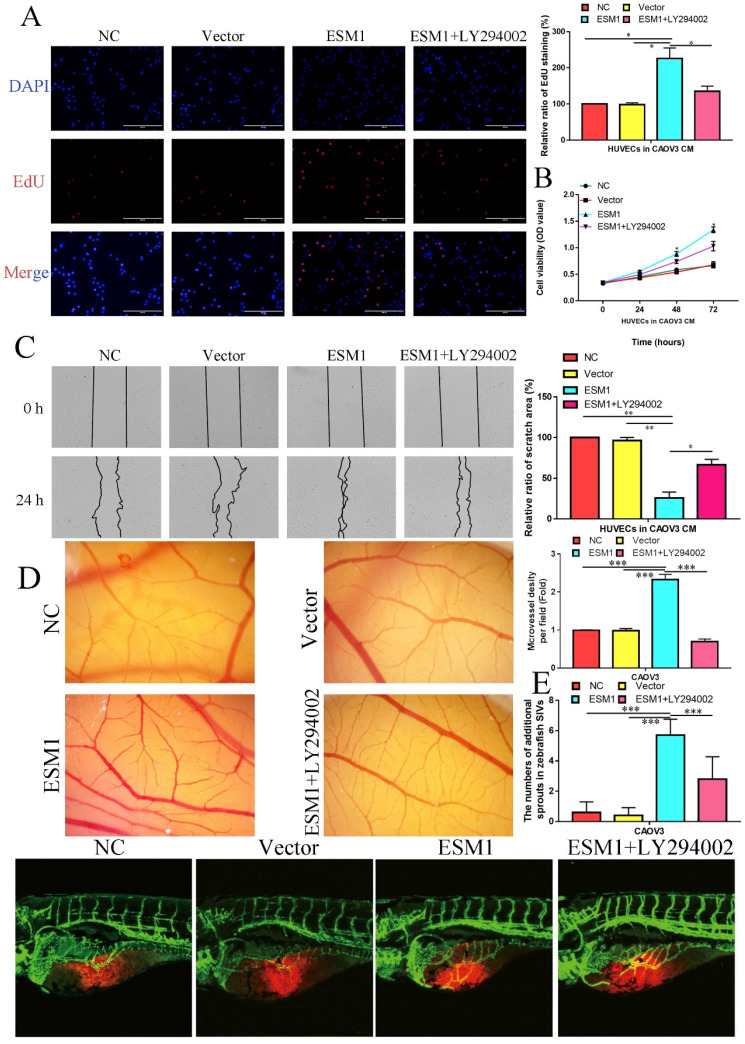
** ESM1 can promote the OC angiogenesis via the Akt pathway *in vitro* and vivo.** The effect of ESM1 or ESM1+LY294002 CAOV3 cell-conditioned medium on HUVECs proliferation ability via EdU analysis **(A)** and MTT assay **(B)**. **C.** The effect of ESM1 or ESM1+LY294002 CAOV3 cell-conditioned medium on HUVECs migration ability via wound healing. **D.** Chicken embryos were incubated with CAOV3-conditioned medium after ESM1 or ESM1+LY294002. **E.** The effects of ESM1 or ESM1+LY294002 CAOV3 cell on angiogenesis in zebrafish model. *P < 0.05, **P < 0.01, ***P < 0.001 indicates statistical significance compared with the control.

EdU analysis also showed that ESM1 could accelerate DNA replication in CAOV3 cells (Figure 11C). Moreover, we found that ESM1 could enhance OC migration and invasion ability (Figure 11D&E), which obviously increased MMP9 but decreased TIMP protein expression (Figure 11F). CAOV3 cells with high levels of ESM1 could induce tumor neovascularization inhibition compared to the NC and vector groups (Figure 11G). Furthermore, these molecular biological effects of ESM1 could be reversed by the Akt inhibitor LY294002 (Figure 11A-G), but ESM1 expression was not affected by the Akt inhibitor LY294002 (Figure 11F). Moreover, we also found ESM1 could enhance the formation of new blood vessels in HUVECs cultured with the CM of CAOV3, which could be rescued by LY294002 (Figure 11H). Further experiments indicated that high levels of ESM1 secreted by ESM1 OE-CAOV3 cell could enhance the proliferation and migration ability of HUVECs (Figure 12A-C). CAM and zebrafish model analysis was used to further confirm the molecular effects of ESM1 on OC angiogenesis *in vivo*, compared to NC and Vector group, ESM1 OE had an obviously increased density and number of new blood vessels formation, while LY294002 showed a remarkedly decrease density and number of micro-vessel formation (Figure 12D&E). These results indicated that ESM1 could promote OC progression via the Akt pathway.

## Discussion

OC is a multiblood vessel solid tumor with a high mortality rate 29. Moreover, there are few special symptoms at the preinvasive stages of OC, and there is currently no screening program. These patients with OC are usually diagnosed late, when the cancer cells have metastasis within the peritoneal cavity and no chance for complete surgical removal. The 5-year survival rate is almost 30% in OC patients diagnosed at a late stage 30. Therefore, efforts to explore therapeutic targets or biomarkers for OC treatment and diagnosis are still of great importance.

Currently, with the expected development of high-throughput sequencing technology, various public databases and bioinformatics algorithms provide a scientific basis and tool in searching hub genes for further exploration of cancer research 31. In our study, we found that 3 genes (CENPH, ESM1 and HIST1H2AE) were closely correlated with the formation, development and progression of OC via the WGCNA network 32 and random survival forest variable screening 10 based on the GSE66957 and GSE54388 datasets. Then, we used CIBERSORT algorithms 33 to quantify immune cell data and further analyzed the correlation between 3 hub genes and immune infiltration, which indicated that ESM1 played a key role in the carcinogenesis of OC.

ESM1, an endocan, is primarily expressed in endothelial cells in human lung and kidney tissues under physiological conditions 34 and is involved in regulating the function of endothelial cells 35. Moreover, recent studies have pointed out that ectopically high levels of ESM1 are observed in multiple cancer types, such as HCC 16, BRCA 20, ESCC 15, PRCA 18, colorectal cancer (CRC) 36, bladder cancer (BCA) 37, HNSC 38, oral squamous cell carcinoma (OSCC) 39, LUAD 17 and gastric cancer 40. We found that ESM1 was also increased in OC tissue samples, which was negatively associated with OC patient OS and positively associated with OC patient PFS. Cui and his colleagues also found that ESM1 was a significant biomarker and negatively correlated with OS for multiple cancer types, including adrenocortical carcinoma (ACC), cervical squamous cell carcinoma and endocervical adenocarcinoma (CESC), ESCC, glioblastoma multiforme (GBM), kidney renal papillary cell carcinoma (KIRP), sarcoma (SARC) and uterine corpus endometrial carcinoma (UCEC) 15. Janowczyk et al. found that ESM1 could be a potential tumor vascular marker for OC patients for prognosis prediction 41. Laloglu and his colleagues also found that malignant ovary and endometrial disease had significantly increased ESM1 levels, which was useful in diagnosing malign or benign disease of the ovary and endometrium 42. These results indicated that ESM1, as an oncogene, played a key role in multiple cancers and might be an effective biomarker for cancer patients, especially in OC. However, the mechanisms contributing to the overexpression of ESM1 remain obscure. In our opinion, the upregulation of ESM1 may be due to DNA methylation (Figure 4D), noncoding RNA regulation, histone acetylation, and the activity of key transcription factors. Moreover, we found that ESM1 expression was significantly correlated with multiple clinical pathological parameters based on IHC staining, including the FIGO stage, lymph node metastasis, and recurrence. Manal Behery et al. found that ESM1 was significantly enhanced in OC samples compared to normal ovary samples, and ESM1 was an independent prognostic marker for OC patients 43. Wang and his colleagues further found that new angiogenesis-related gene (ARG) signatures (ESM1, CXCL13, TPCN2, PTPRD, FOXO1, and ELK3) performed moderately well in OC prognostic predictions 44. These results indicated that ESM1 induces poor prognosis for OC patients by inducing angiogenesis, lymph node metastasis, and recurrence. However, all these hypotheses require further experimental verification.

Compared to NC- or vector-transfected OC cells, ESM1 knockdown OC cells had lower proliferation and apoptosis escape abilities. Previous studies have reported that ESM1 could accelerate myeloid leukemia cell 45, BRCA cell 20 and NSCLC cell 17 proliferation, as well as promote the apoptosis escape of CRC cells 46, myeloid leukemia cells 45, and BCA cells 47. Moreover, the primary molecular mechanisms were that ESM1 could activate the Akt 20, NF-κB 46, and MAPK 48 signaling pathways. In addition, multiblood vessel solid tumors are another feature of OC 29. We confirmed the effect of ESM1 knockdown on angiogenesis by decreasing the level of VEGF in OC cell lines. A previous study indicated that a positive feedback loop between VEGF and ESM1 was involved in the progression of BCA 49. ESM1 can also interact with multiple angiogenic molecules, such as HGF/SF and FGF, to form a positive feedback loop, which further induces the proliferation and tubular structure formation of vascular endothelial cells 34. Furthermore, previous studies suggested that VEGF could induce the activation and formation of lymphatic vessels 50, 51. ESM1 could induce proliferation and migration via VEGF to induce lymphangiogenesis and cancer metastasis 51. In our study, we also found that ESM1 knockdown could suppress OC cell migration and invasion. The essential elements of migration and invasion are part of the cell metastasis ability, which is mainly responsible for the primary mortality of cancers 52. In the progression of cancer cell migration and invasion, matrix metalloproteinase (MMP) expression is an important cause of cancer cells dropping from the primary focus and spreading to new tissues 53. A series of studies indicated that ESM1 could accelerate migration and invasion in multiple cancer types, such as HCC, HNSC, CRC and GC 46, 48, 54, 55. We also found that the migration and invasion molecular mechanism of ESM1 in OC progression was similar to that in these cancer types. These results both indicated that ESM1 played a key role in cancer metastasis.

Based on our bioinformatics analysis (Figure 3C) and experimental validation (Figure 9A), we confirmed that ESM1 could accelerate OC carcinogenesis (proliferation, apoptosis, metastasis, and angiogenesis) via the Akt/mTOR pathway, which has been demonstrated to be a significant cancer-promoting signaling pathway. In more recent studies, the Akt/mTOR signaling pathway was shown to be involved in OC cancer cell proliferation, apoptosis, metastasis, and angiogenesis 56. Combined with the results of our bioinformatic analysis and experimental validation, these results indicated that ESM1 can drive the activation of the Akt/mTOR pathway to promote the occurrence and progression of OC.

## Conclusions

We first systematically elucidated the expression, function and molecular mechanism of ESM1 in OC progression. We confirmed that ESM1 could increase proliferation, apoptosis escape, angiogenesis, migration and invasion by regulating the Akt/mTOR pathway. In summary, this work further confirmed that ESM1 could be a diagnostic marker and potential therapeutic target for OC patients.

## Supplementary Material

Supplementary figures.

## Figures and Tables

**Table 1:** The expression of ESM1 in OC based on IHC staining

Groups	Patient number	ESM1 level		X^2^	*P* value
		Low	High		
**Diagnostic category**				48.62	
Normal	15	13	2		
Paracancer tissues	37	22	15		0.0581^a^
Ovarian cancer	60	4	56		<0.0001^a,b^

^a^ Compared to normal tissue; ^b^ Compared to paracancer tissues.

**Table 2:** The relationship between clinical pathological parameters and ESM1 based on IHC staining

Pathological parameters	patient number	ESM1 level		X^2^	*P* value
		Low	High		
**Age**				0.01913	0.8900
<50	28	2	26		
≥50	32	2	30		
**CA 125 (IU/ml)**				1.694	0.193
≤500	17	0	17		
>500	43	4	39		
**FIGO stage**				21.43	<0.0001
I	3	2	1		
II	15	2	13		
III	31	0	31		
IV	11	0	11		
**Lymph node metastasis**				4.596	0.032
Yes	43	1	42		
No	17	3	14		
**Recurrence**				14.1	0.0002
Yes	52	1	51		
No	8	3	5		

## Abbreviations

OC: Ovarian cancer
ESM1: Endothelial cell-specific molecule 1
DEGs: Differentially expressed genes
PD1: Programmed cell death protein 1
EGFR: Epidermal growth factor receptor
WGCNA: Weighted gene coexpression network analysis
RWR: Random walking with restart
GEO: Gene Expression Omnibus
GO: Gene Ontology
KEGG: Kyoto Encyclopedia of Genes
PPI: Protein-protein interaction
BLCA: Bladder urothelial carcinoma
BRCA: Breast invasive carcinoma
COAD: Colon adenocarcinoma
CHOL: Cholangiocarcinoma
LUAD: Lung adenocarcinoma
ESCA: Esophageal carcinoma
HNSC: Head and neck squamous cell carcinoma
KIRC: Kidney renal clear cell carcinoma
KICH: Kidney chromophobe
LIHC: Liver hepatocellular carcinoma
PRAD: Prostate cancer
STAD: Stomach adenocarcinoma
THCA: Thyroid carcinoma
UCEC: Uterine corpus endometrial carcinoma
CAM: Chorioallantoic membrane assay
CM: Conditioned medium
GSVA: Gene set variation analysis
TMB: Tumor mutational burden
OS: Overall survival
PFS: Progression-free survival
OE: Overexpression
CRC: Colorectal cancer
BCA: Bladder cancer
OSCC: Oral squamous cell carcinoma
ACC: Adrenocortical carcinoma
CESC: Cervical squamous cell carcinoma and endocervical adenocarcinoma
GBM: Glioblastoma multiforme
KIRP: Kidney renal papillary cell carcinoma
SARC: Sarcoma
UCEC: Uterine corpus endometrial carcinoma
ARG: Angiogenesis-related gene
MMP: Matrix metalloproteinase
