# Supplementary material for: Validation of ESM1 Related to Ovarian Cancer and the Biological Function and Prognostic Significance
Source: Int J Biol Sci. 2023 Jan 1;19(1):258–80. doi: 10.7150/ijbs.66839 (PMC9760436; doi:10.7150/ijbs.66839)
Supplement: Supplementary file 1 — Supplementary figures. [file ijbsv19p0258s1.pdf]

Supplement Figure 1

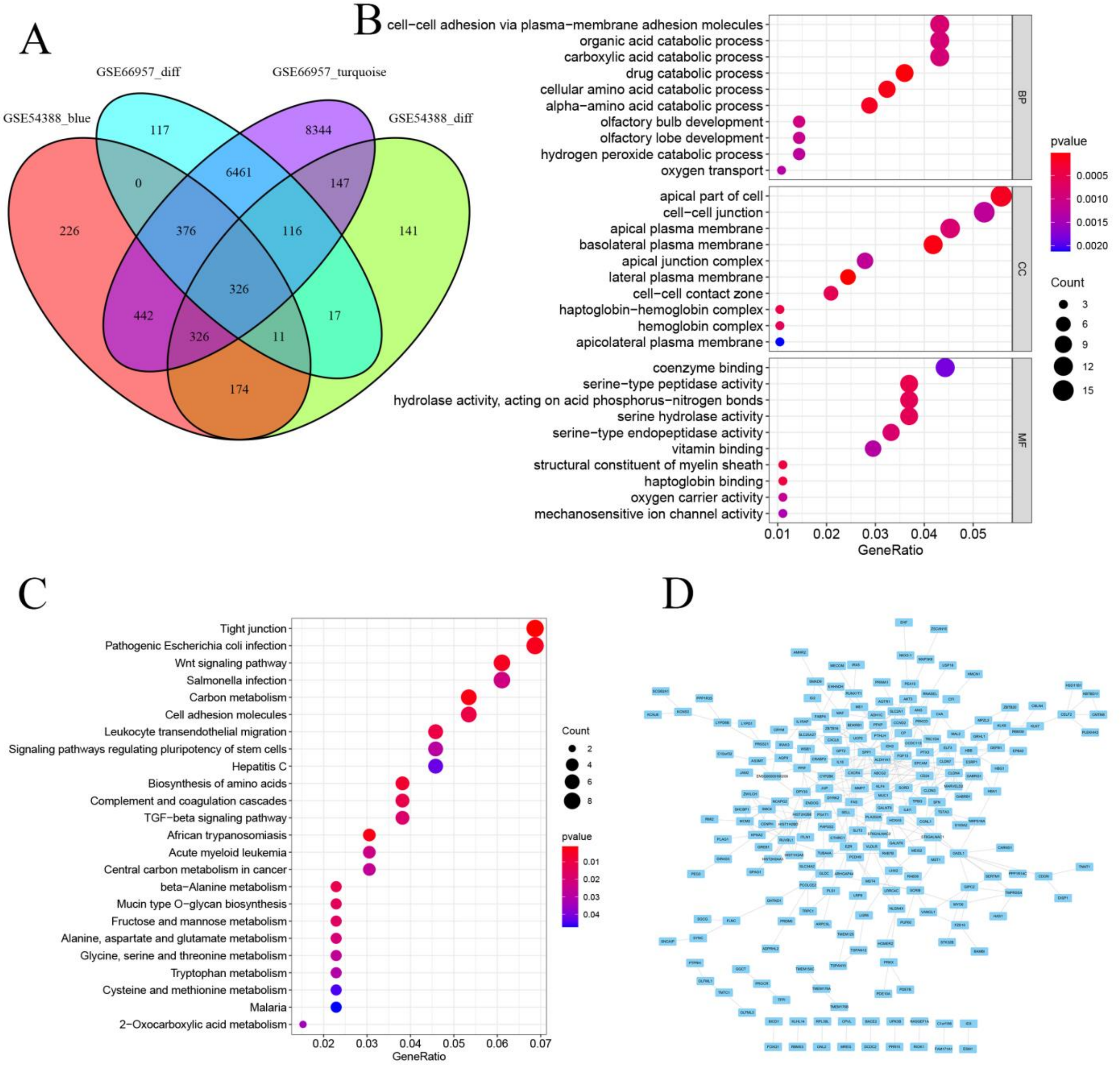

Supplement Figure 1. Identification of the hub genes and PPI network construction. A. Hub genes were identified based on the overlap among the hub modules of the GSE66957 turquoise module, GSE54388 blue module, GSE66957 DEGs, and GSE54388 DEGs. B. GO enrichment for hub genes. C. The KEGG pathway enrichment for hub genes. D. PPI network construction based on hub genes.

Supplement Figure 2

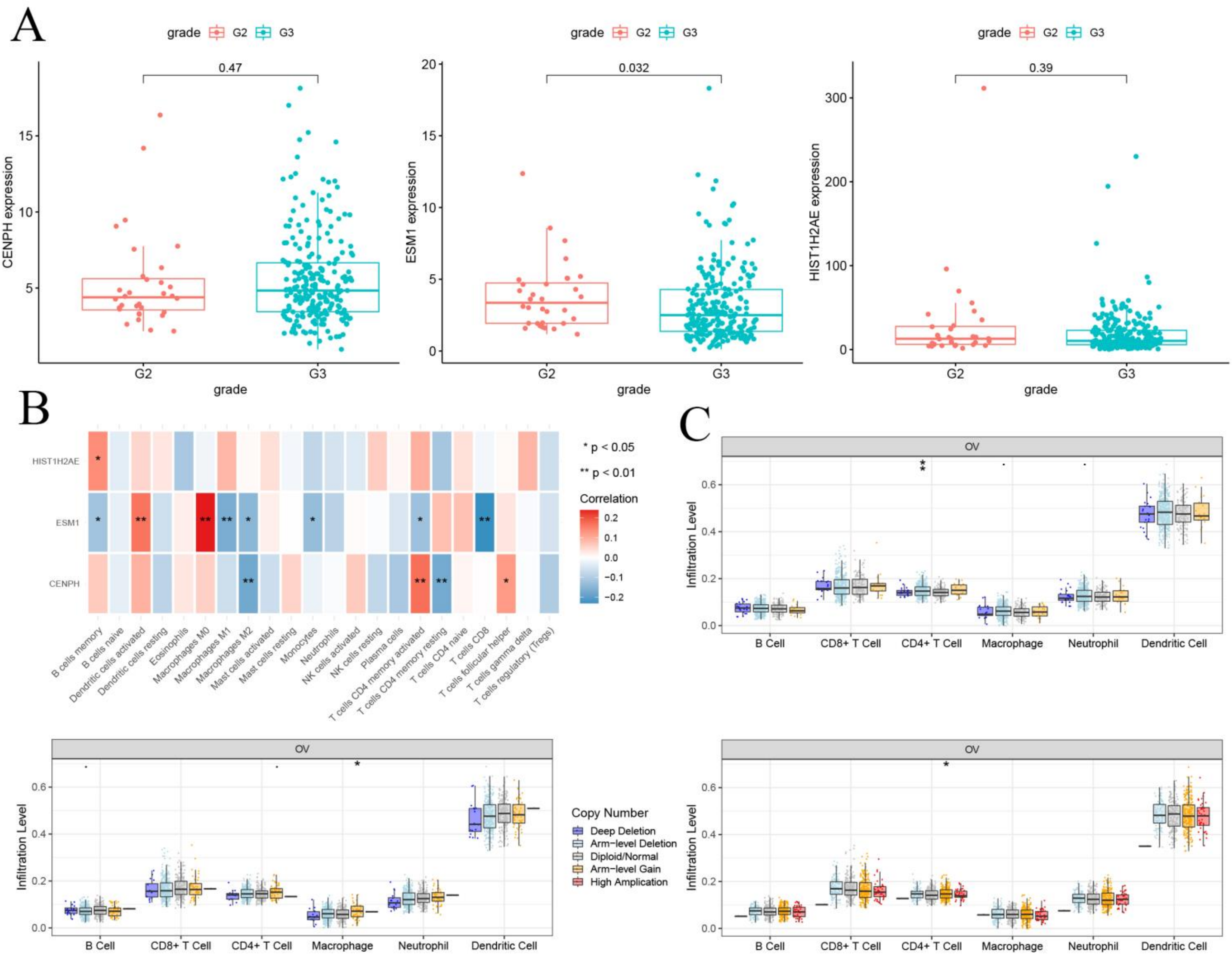

Supplement Figure 2. Relationship between key genes and immune infiltration. A. Relationship between expression and pathology grade for ESM1, CENPH and HIST1H2AE. B. The mRNA level profile of 3 key genes in multiple immune cell types by the TCGA database. C. CNV affecting the distribution of 3 key genes based on the TIMER database.

Supplement Figure 3

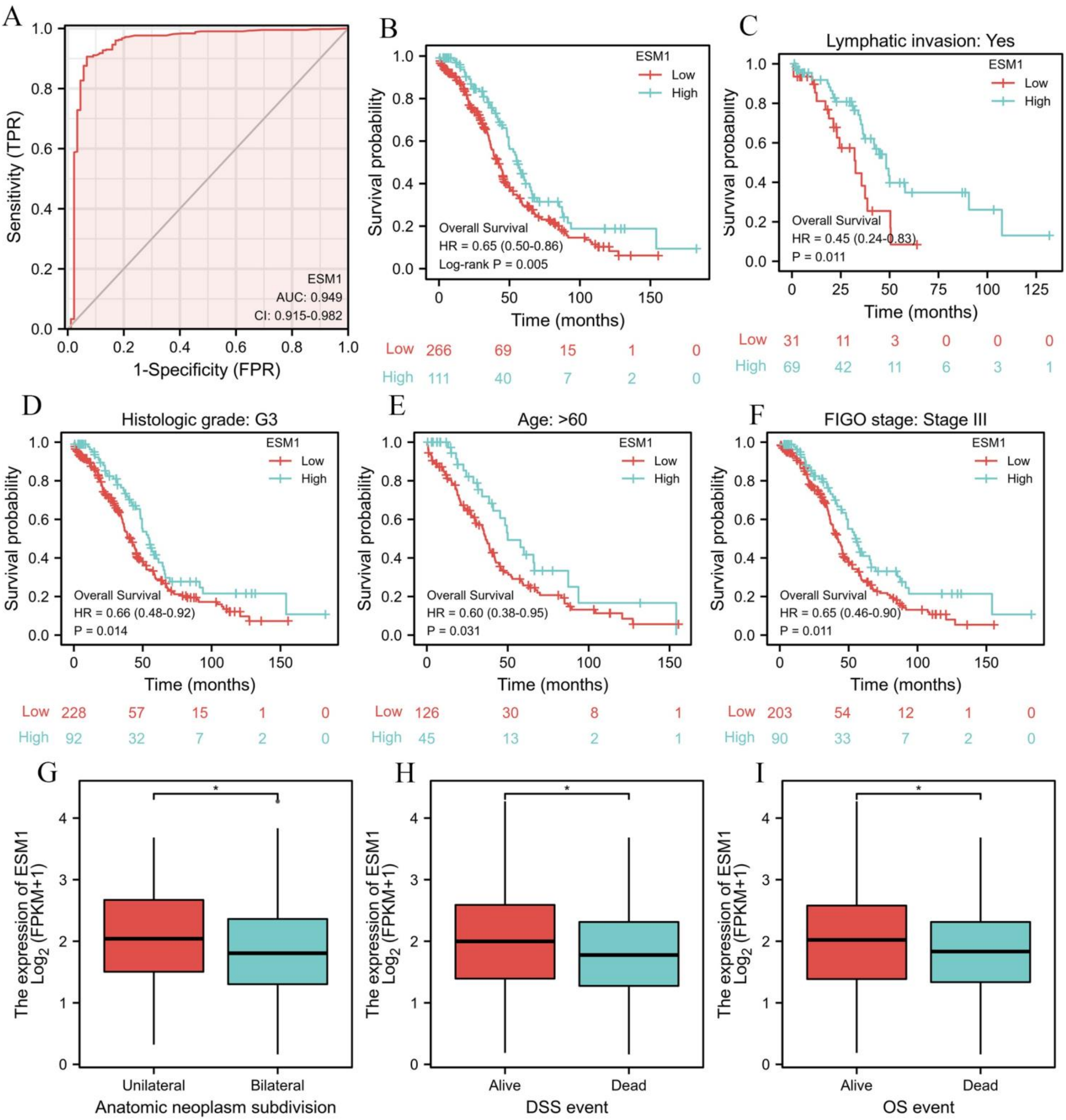

Supplement Figure 3. The significance of ESM1 in OC progression based on TCGA database. A. The diagnostic value of ESM1 in OC patients based on TCGA database. B. The overall survival values of ESM1 in OC patients with lymphatic invasion (C), histologic grade G3 (D), Age>60 (E), or FIGO stage III (F). The expression of ESM1 in different anatomic neoplasm subdivision (G). DSS event (H), or OS event (I).

Supplement Figure 4

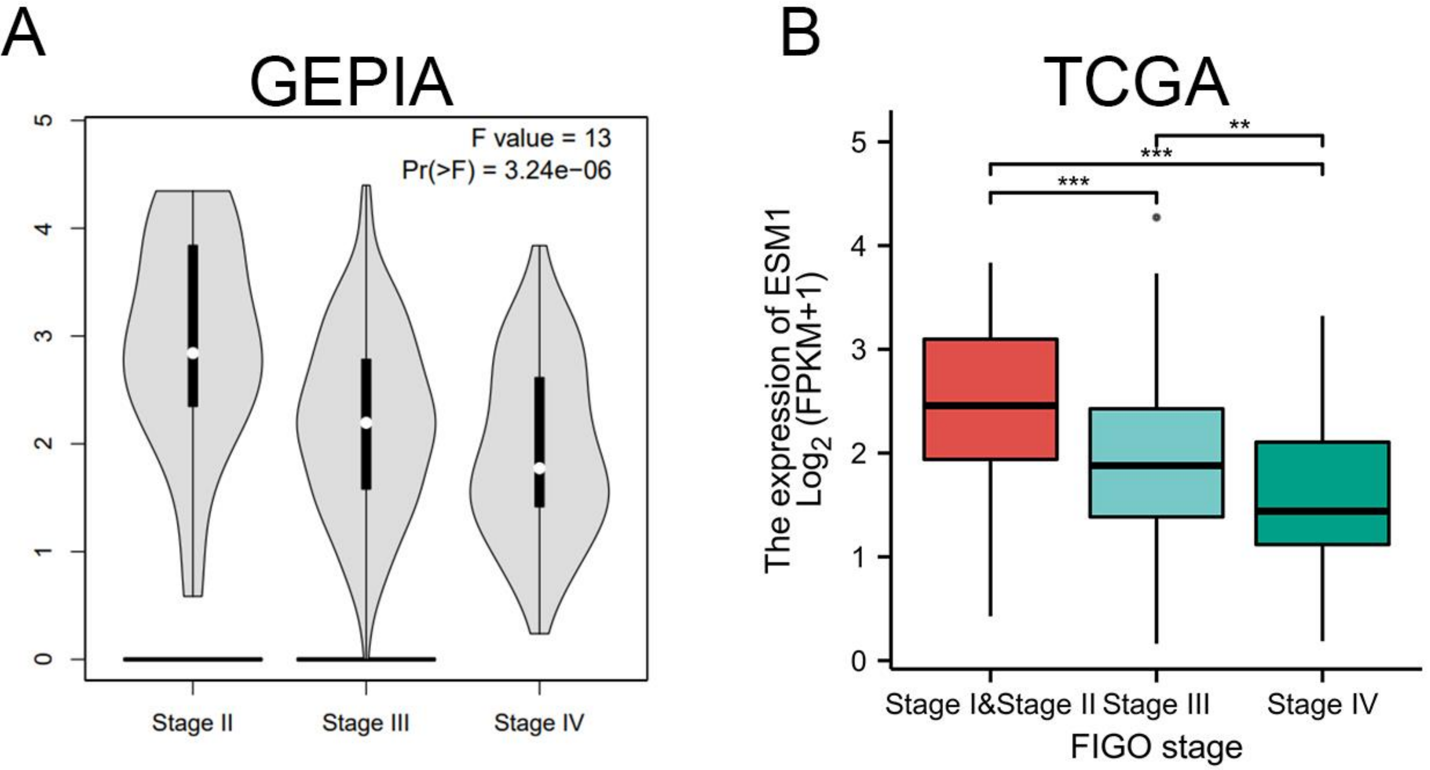

Supplement Figure 4. The expression of ESM1 in OC patients at different stages. The ESM1 mRNA level of OC patients at different stages based on GEPIA database (A) and TCGA database (B). **P < 0.01, ***P < 0.001 indicates statistical significance compared with the control.
